# Factors Related to the Post-operative Recurrence of Atypical Meningiomas

**DOI:** 10.3389/fonc.2020.00503

**Published:** 2020-04-15

**Authors:** Wu Ye, Tang Ding-Zhong, Yang Xiao-Sheng, Zhan Ren-Ya, Li Yi

**Affiliations:** ^1^Department of Neurosurgery, Tongde Hospital of Zhejiang Province, Hangzhou, China; ^2^Department of Neurology, Jinshan Branch of Shanghai Sixth People's Hospital, Shanghai, China; ^3^Department of Neurosurgery, School of Medicine, Ninth People's Hospital, Shanghai Jiao Tong University, Shanghai, China; ^4^Department of Neurosurgery, The First Affiliated Hospital of Medical School of Zhejiang University, Hangzhou, China

**Keywords:** atypical meningiomas, prognosis, recurrence, clinicopathological characteristics, predictive factors, extent of resection, tumor invasiveness

## Abstract

**Aim:** This study aimed to investigate the relationship between clinicopathological characteristics of atypical meningiomas (AM) and its post-operative recurrence.

**Materials and Methods:** The clinicopathological characteristics and findings from follow up were retrospectively reviewed and compared between AM and benign meningioma (BM) patients. Univariate and multivariate analyses were employed to identify the factors related to the post-operative recurrence of AM.

**Results:** More BM patients were females and received complete resection; the recurrence rate was significantly lower in BM patients as compared to AM patients. The progesterone receptor (PR), E-cadherin protein (E-Ca) and β-catenin positive rates and Ki67 labeling index were significantly different between two groups. Univariate analysis showed the age, tumor size, tumor invasiveness, E-Ca expression, and extent of resection were related to the post-operative recurrence of AM. However, multivariate analysis showed only the extent of resection and tumor invasiveness were the independent factors associated with the post-operative recurrence of AM.

**Conclusions:** The extent of resection and tumor invasiveness are related to the post-operative recurrence of AM. To improve the surgical procedures to maximize the tumor resection is important to improve the prognosis of AM patients.

## Introduction

Meningiomas, a tumor of meningothelial cell origin, are the second most common intracranial tumor and account for about 24–30% of intracranial tumors (1–3). According to the World Health Organization classification system, meningiomas are classified into grade I (typical), grade II (atypical), and grade III (anaplastic) tumors. Most meningiomas are benign (grade I), and atypical meningiomas (AM) account for about 5–7% of meningiomas.

Surgery is a major treatment for meningiomas. The benign meningiomas have a relatively low risk of recurrence (~10%) after complete resection, but AM and anaplastic meningiomas are characteristically more aggressive in nature and associated with higher recurrence risks (29–52% and 50–94%, respectively) (4). Benign meningiomas (BM) seem to be related to estrogen levels and are more common in women, but AM and anaplastic meningiomas are more common among men and also seem to have a greater predilection for the cerebral convexities. Some studies have investigated the prognostic factors of AM. Zaher et al. reported that age (<50 years) and total surgical excision were independent prognostic factors for survival and radiotherapy could reduce the post-operative recurrence (5). There is evidence showing that age, male gender, extent of surgical resection, and higher MIB-1 (a commonly used monoclonal antibody that detects the Ki-67 antigen) labeling index influence the prognosis of AM patients, and postoperative radiotherapy fails to provide long-term tumor control (6, 7). In a Korea study, results showed the overall survival was not related to the patient age, gender, tumor location, Ki-67 (a cellular marker for proliferation), Simpson grade, and treatment (8). In patients with skull base AM, the age of disease onset and MIB-1 index were found to be independent prognostic factors of clinical outcome, and adjuvant radiotherapy was recommended to reduce recurrence regardless of the extent of surgical resection (9). Ros-Sanjuan et al. found the extent of excision was the only predictor of post-operative recurrence (10), but Streckert et al. found the brain invasion was also found to be associated with the post-operative recurrence (11). These differences might be ascribed to the age, race, sample size, tumor location, dose of radiotherapy, and other factors. Currently, little is known about the factors related to the post-operative recurrence in AM patients in China mainland.

In the present study, we retrospectively investigated 30 patients diagnosed with AM in two clinical centers, and the clinicopathological characteristics and findings from follow up were collected and compared between AM patients and benign meningioma (BM) patients as controls. Furthermore, the relationships of these factors with post-operative recurrence were explored in AM patients, aiming to provide evidence on the clinical management of AM.

## Materials and Methods

### Patients

A total of 1,068 patients were diagnosed with meningioma according to the 2007 WHO classification in the Department of Neurosurgery of two hospitals (The First Affiliated Hospital of Medical School of Zhejiang University and Tongde Hospital of Zhejiang Province). Among these patients, 37 were diagnosed with AM (3.4%), of whom 30 had complete medical record and received follow up. All these patients were initially diagnosed with AM and did not received surgery or radiotherapy before study. The pathological diagnosis of AM was confirmed by two experienced pathologists (12). In addition, 30 patients diagnosed with BM (WHO grade I) and having complete clinical record were included as controls: they were pathologically diagnosed with BM in the same period; they had no severe heart, liver, and liver disease; there was no metastasis; the age ranged from 18 to 80 years. This was a retrospective study and approved by the Institutional Review Board of two hospitals.

### Data Collection

The clinicopathological characteristics were collected by reviewing the medical record: age, gender, surgical findings, imaging findings, presence of and time to post-operative recurrence, managements after recurrence, and survival status. The maximal tumor diameter (cm) was determined on MRI or CT.

### Immunohistochemistry

The surgically collected tissues were embedded in paraffin, and 4-μm sections were obtained, followed by immunohistochemistry with two-step Envision method. The surrounding normal brain tissues were used as a positive control, and phosphate buffered solution (PBS) was used instead of primary antibody in the negative control. Following proteins were detected by immunohistochemistry: progesterone receptor (PR), Ki-67, E-cadherin protein (E-Ca) and β-catenin, and following antibodies were used: mouse anti-human PR, mouse anti-human Ki-67 (Long-Island Diagnostic Reagent Co., Ltd), mouse anti-human E-Ca, mouse anti-human β-catenin, and anti-mouse secondary antibodies (DAKO company). In the immunohistochemistry, 3,3'-diaminobenzidine (DAB), hematoxylin and 0.1% hydrochloric acid (HCL) were used, and mounting was done with neutral gum. After immunohistochemistry, sections were observed under a light microscope (OLYMPUS, Japan).

### Pathological Assessment

Normally, E-Ca is expressed on the cell membrane, and loss of E-Ca expression on the cell membrane is abnormal. Normally, β-catenin is expressed on the cell membrane and in the cytoplasm, and nuclear expression of β-catenin is abnormal. Cells with yellowish-brown nucleus were regarded positive after immunohistochemistry for PR. Cells with yellow or brown nucleus were regarded positive after immunohistochemistry for Ki67.

Two experienced pathologists scored the pathological findings according to the semi-quantitative immunoreactive scoring (IRS) method: (1) staining intensity ( ×400): no staining, 0; light yellow, 1, yellowish-brown, 2; brown, 3; (2) counting of positive cells: five fields were randomly selected at ×200, and a total of 200 tumor cells were counted in each field. The proportion of positive cells was determined as follows: <5%, 0; 5–25%, 1; 26–50%, 2; 51–75%, 3; >75%, 4. The final score was the product of above two scores and classified as – (0), – (1–2), ++ (3–6) and +++ (>6). Positive staining refers to +, ++, and +++, and negative staining refers to –. Ki67 labeling index (Ki67LI) was calculated as follow: number of positive cells / total cells ×100%.

### Determination of Edema Index

The edema index (EI) was defined as the edema/tumor volume ratio as previously reported (13): (V_edema_–V_tumor_)/V_tumor_. The maximal length, width and height of the tumor or edema were measured and the tumor volume was calculated as follow: V = (length × width × height)/2. The peritumoral brain edema was scored as follow: 0, EI = 0 (no peritumoral brain edema); 1, 0 < EI ≤ 1 (mild peritumoral brain edema); 2, 1 < EI ≤ 2 (intermediate peritumoral brain edema); 3, EI>2 (severe peritumoral brain edema).

### Assessment of Tumor Invasiveness

The tumor invasiveness was mainly assessed macroscopically and microscopically.

#### Brain Invasion

Invasion of the tumor into the brain parenchyma was deemed present when any of the following criteria were met after we performed a standard sampling protocol: (1) the brain–tumor interface was observable and showed invasion of tumor into the brain on hematoxylin-eosin (HE) staining; (2) a sample of tumor was available containing brain tissues within it that displayed reactive astrocytosis or neuronal degeneration; or (3) reactive astrocytes within the tumor were revealed using standard glial fibrillary acidic protein (GFAP) immunostaining.

#### Dural Invasion

(1) the basal dura mater was invaded by tumor cells on HE staining; (2) tumor cells invaded the dura mater and cause damage; (3) tumor cells invaded the sagittal sinus or contralateral falx cerebri; (4) The area of thickening dura mater close to the tumor was one or more times that of the tumor.

#### Skull Invasion

(1) The tumor invasion caused local skull deformation or defect; (2) The tumor was adherent to the skull after penetrating the dura mater, and pathological examination showed tumor cell infiltration or tumor nest formation in the bone.

#### Multiple-Site Invasion

The tumor cells invaded two or more tissues.

### Determination of Extent of Resection

The extent of resection was determined according to the Simpson Grading scale: grade I, macroscopically complete tumor resection with removal of affected dura and underlying bone; grade II, macroscopically complete tumor resection with coagulation of affected dura only; grade III, macroscopically complete tumor resection without removal of affected dura or underlying bone; grade IV, subtotal tumor resection; grade V, decompression with or without biopsy (14).

### Statistical Analysis

Statistical analysis was performed with SPSS version 17.0 (SPSS Inc., Chicago, IL, USA). Quantitative data are expressed as mean ± standard deviation (SD) and compared with *t*-test; categorical data were compared with Chi square test. Univariate analysis was done with Kaplan-Meier method to evaluate the relationship of clinicopathological factors (gender, age, tumor diameter, tumor location, peritumoral edema, tumor invasiveness, Ki-67 LI, E-ca positive rate, β-catenin positive rate, PR positive rate, extent of surgical resection, and post-operative radiotherapy) with post-operative recurrence. The independent factors related to the post-operative recurrence of AM were determined by using the multivariate Cox regression analysis. A value of *P* < 0.05 was considered statistically significant.

## Results

### Clinical Characteristics of Patients in the AM Group and BM Group

In the AM group, there were 19 females and 11 males, and the mean age was 58.6 ± 16.6 years (range: 20–81 years); 46.7% (14/30) of patients were younger than 60 years. In the BM group, there were 26 females and 4 males, and the mean age was 53.6 ± 14.6 years (range: 5–74 years); 63.3% of patients were younger than 60 years (Table 1). Significant difference was observed in the gender between AM group and BM group, but there was no marked difference in the age. The skull base, convexity, and parasagittal meningiomas were found in 7, 17, and 6 patients, respectively, in the AM group, and 11, 13, and 6 patients, respectively, in the BM group, showing no pronounced difference between two groups in the tumor localization. The mean tumor diameter was 5.4 ± 1.7 cm (range: 1.5–10 cm) in the AM group and 4.4 ± 2.0 cm (range: 1.1–11 cm), there was marked difference in the tumor size between them, and more patients in the AM group had the tumor larger than 5 cm in the diameter.

**Table 1:** Clinicopathological characteristics of AM patients and BM patients.

**Variables**		**AM (*n* = 30)**	**BM (*n* = 30)**	***P***
Gender				
	M	11 (36.7)	4 (13.3)	^*^P < 0.05
	F	19 (63.3)	26 (86.7)	
Age (years)				
	Mean	58.6 (20–81)	53.6 (5–74)	*P* > 0.05
	≤ 60	14 (46.7)	19 (63.3)	
	>60	16 (53.3)	11 (36.7)	
Location				
	Skull base	7	11	*P* > 0.05
	convexity	17	13	
	parasagittal	6	6	
Tumor diameter (cm)				
	Range	5.4 (1.5–10)	4.2 (1.1–11)	^*^P < 0.05
	≤ 5	14 (46.7)	23 (76.0.7)	
	>5	16 (53.3)	7 (23.3)	
Peritumoral edema				
	Mild (0, 1)	16 (53.3)	13 (43.3)	*P* > 0.05
	Severe (2, 3)	14 (46.7)	17 (56.7)	
Extent of surgical resection				
	Total	18	26	^*^P < 0.05
	Subtotal	12	4	
Radiotherapy	Yes	7	3	*P* > 0.05
	No	23	27	
Recurrence	Yes	12	2	^*^P < 0.05
	No	18	28	
Death	Yes	2	0	*P* > 0.05
	No	28	30	
Invasiveness	Meninges	30 (100%)	25 (83.3%)	^**^P < 0.01
	Brain	12 (40%)	2 (6.7%)	
	Skull	11 (36.7%)	2 (6.7%)	
	Multiple	17 (56.7%)	4 (13.3%)	
β-catenin	Positive	13	27	X^2^=14.70, ^*^P < 0.01
	Negative	17	3	
PR	Positive	14	24	X^2^ = 7.18, ^*^P < 0.01
	Negative	16	6	
E-Ca	Positive	13	26	X^2^ = 12.38, ^*^P < 0.01
	Negative	17	4	
Ki67	Positive	13	1	X^2^ = 13.42, ^*^P < 0.01
	Negative	17	29	

*AM, atypical meningioma; BM, benign meningioma; E-ca, E-cadherin protein; F, female; M, male; PR, progesterone receptor*.

* *P < 0.05*,

** *P < 0.01: AM group vs BM group*.

In addition, mild and severe peritumor edemas were noted in 16 and 14 patients, respectively, in the AM group and 13 and 17 patients, respectively, in the BM group, showing no significant difference. Simpson grade I and II were defined as total resection, and Simpson grade III, IV, and V as sub-total resection. As shown in the Table 1, the percentage of patients receiving total resection was 86.7% in the BM group (26/30), which was significantly higher than in the AM group (60.0%; 18/30; *P* < 0.05).

In the AM group, seven patients received post-operative radiotherapy, of whom five underwent gamma knife surgery and two received whole brain radiotherapy. In the BM group, three patients received post-operative radiotherapy (all with gamma knife surgery). Patients in both groups did not receive post-operative chemotherapy. There was no marked difference in the proportion of patients receiving post-operative radiotherapy although it in the AM group was slightly higher than in the BM group (*P* > 0.05).

### Findings From Follow Up

In the AM group, 30 patients received complete follow up for a median of 34 months (range: 3–69 months), 12 developed recurrence and two died. In the BM group, 30 patients received complete follow up for a median of 32.5 months (range: 18–80 months), two developed recurrence and none died. The recurrence rate in the AM group (40%; 12/30) was significantly higher than in the BM group (6.7%; 2/30) (*P* < 0.05).

### Immunohistochemical Findings

Fourteen and twenty-four patients were positive to PR in the AM group and BM group, respectively, showing marked difference between them (*P* < 0.01). Positive E-Ca expression was found in 13 and 26 patients in the AM group and BM group, respectively, showing dramatic difference (*P* < 0.01). 13 and 27 patients were positive to β-catenin in the AM group and BM group, respectively, showing marked difference (*P* < 0.01). The Ki67 LI was 8 ± 4.32% in the AM group and 2.83 ± 1.77% in the BM group, and significant difference was noted in the Ki67 LI between them. Moreover, Ki67 LI ≥8% was found 13 patients in the AM group, but only one patient in the BM group (*P* < 0.01).

### Univariate Analysis of Factors Related to the Post-Operative Recurrence

Results showed age, tumor size, tumor invasiveness, and extent of resection were positively related to the post-operative recurrence. The slightly increased recurrence rate in the patients receiving post-operative radiotherapy seemed to be associated with the use of sub-total resection in these patients. In addition, the recurrence rate was also significantly different between patients with triple invasion and remaining patients (Table 2).

**Table 2:** Univariate analysis of clinicopathological factors with post-operative recurrence in AM patients.

**Variables**	***n***	**Recurrence (%)**	**Mean**	**Median (range)**	***P***
Age (years)			58.6	65 (20–81)	^*^P = 0.01
0-60	14	2 (14.3)			
>60	16	10 (62.5)			
Gender					*P* = 0.06
M	11	2 (18.1)			
F	19	10 (52.6)			
Location					*P* > 0.05
Convexity	17	6 (35.3)			
Parasagittal	6	2 (33.3)			
Skull base	7	4 (57.1)			
Maximal diameter (cm)			5.4	5.2 (1.5–10.0)	^*^P = 0.01
0–5.0	14	2 (14.3)			
>5.0	16	10 (62.5)			
Peritumoral edema					0.28
Mild	16	6(37.5)			
Severe	14	6 (42.8)			
Tumor invasiveness					^*^P = 0.02
Single or double invasion	24	7 (25.9)			
Triple invasion	6	5 (83.3)			
Extent of resection					^*^P = 0.00
Total	18	2 (11.1)			
Subtotal	12	10 (83.3)			
Radiotherapy					*P* > 0.05
Yes No	7 23	3 (42.9)9 (39.1)			
Ki67			8.0%	7.0% (3–25%)	*P* = 0.12
< 8%	17	5 (29.4)			
≥8%	13	7 (53.8)			
E-ca					^*^P = 0.02
Negative	17	10 (58.8)			
Positive	13	2 (15.4)			
β-catenin					*P* = 0.20
Negative	17	8 (60.0)			
Positive	13	4 (20.0)			
PR					*P* = 0.28
Negative	16	6 (37.5)			
Positive	14	6 (42.9)			

*AM, atypical meningioma; E-ca, E-cadherin protein; F, female; M, male; PR, progesterone receptor*.

* *P < 0.05: among subgroups*.

In addition, E-Ca expression was negatively related to the recurrence rate in the AM group. Although more patients with Ki67 LI ≥8% or being negative to β-catenin had higher recurrence rate, no statistical significance was observed. There was no significant relationship between PR and post-operative recurrence in the AM group (Table 2).

### Multivariate Analysis of Factors Related to the Post-Operative Recurrence

Multivariate analysis showed the extent of resection (*P* = 0.029 < 0.05) and tumor invasiveness (*P* = 0.045 < 0.05) were closely related to the post-operative recurrence of AM (Table 3).

**Table 3:** Multivariate analysis of factors related to the post-operative recurrence in AM patients.

**Variables**	**B**	**SE**	**Wald**	**df**	**sig**	**Exp(B)**
Age	0.354	0.322	1.205	1	0.272	1.425
Gender	0.219	4.368	0.003	1	0.960	1.245
Location	1.007	1.093	0.849	1	0.357	2.739
Maximal diameter	−2.295	1.332	2.972	1	0.085	0.101
Peritumoral edema	2.392	2.105	1.291	1	0.256	10.934
Tumor invasiveness	−5.759	2.868	4.032	1	0.045	0.003
Extent of resection	7.605	3.492	4.742	1	0.029	2008.067
Radiotherapy	11.480	7.868	2.129	1	0.145	96717.926
Ki67	0.034	0.233	0.022	1	0.882	1.035
E-ca	5.934	3.894	2.323	1	0.128	377.752
β-catenin	2.228	2.283	0.953	1	0.329	9.286
PR	−0.583	1.926	0.092	1	0.762	0.558

*AM, atypical meningioma; E-ca, E-cadherin protein; PR, progesterone receptor*.

## Discussion

Meningiomas are slow-growing, well-circumscribed tumors arising from the arachnoid cap cells of the dura mater. Most meningiomas are benign, which corresponds to the WHO grade I, and AM (WHO grade II) are reported in 5–7% of all cases (3, 4). In the present study, we first compared some clinical characteristics between AM and BM patients. The incidence of meningiomas rises with age and they are most common in sixth and seventh decade of life. In addition, it has been reported that there is a marker female predilection with the female to male ratio of 3:2 to 2:1 (3–5). However, there is evidence showing that BM is more common in women, which seems to be linked to the estrogen levels, and AM and anaplastic meningiomas are more common in males, which might be ascribed to the higher proliferation indices in male meningioma patients (15). In the present study, the female to male ratio was 1.7: 1 in the AM group, and 6.5: 1 in BM group, which were consistent with the above findings and suggested the female predominance in BM. In addition, about 53.3% of AM patients and about 36.7% of BM patients were older than 60 years. This was inconsistent with previously reported, which might be ascribed to the small sample size. Moreover, there was no marked difference in the age between two groups. The distribution of tumor location and the EI were similar between two groups, but AM was significantly larger than BM in our study, and significantly more BM patients received total resection as compared to AM patients (86.7 vs. 60%). These may be related to the more aggressive nature of AM (15). Although more AM patients received post-operative radiotherapy, significant difference was not observed between them.

Surgery remains a mainstay of treatment of meningiomas. The microsurgical removal is a preferred treatment for meningiomas when feasible and with acceptable clinical risk. After surgery (especially complete resection of the tumor), BM patients usually have a favorable prognosis, but AM patients often have the risk for post-operative recurrence despite gross-total resection with removal of involved dura and bone and even after substantial time from the initial surgery, which significantly affects the post-operative survival. The prognosis (such as survival time and recurrence) is closely related to the histological grade of meningiomas and the surgical methods. In a large-scale study, according to the histological grade, the estimated 5 years survival was only 70% in patients with benign tumors, 75% for AM and 55% for patients with anaplastic tumors (16); in another population-based studies, the reported 5-year survival rate was near 90% (17). Berrino et al. reported, the 5 years recurrence rate was 3% in BM, 38% in AM and 78% in anaplastic meningiomas after complete resection (18). In the study of Ostrom et al., the 5-year recurrence rate was about 50% for grade II tumors and 90% for grade III tumors (19). Our results also showed significant difference in the recurrence rate between AM group and BM group (40 vs. 6.7%). In the present study, the overall recurrence rate was 40% within a median of 34 months; 60% of AM patients received complete resection and the recurrence rate was 11.1% in patients receiving complete resection. Currently, the recurrence rate varies among available studies, which might be partially related to the definition of recurrence, surgical methods and therapeutic strategies (3).

Studies have investigated the predictors of post-operative recurrence of meningiomas. The extent of resection, tumor pathological features, tumor location and size, tumor invasion, peritumoral edema, age, and gender have been found to be associated with the post-operative recurrence of meningiomas (20). However, little is known about the factors related to the post-operative recurrence of AM in Chinese population. In the present study, we investigated the relationships of some clinicopathological characteristics of AM with the post-operative recurrence. The univariate analysis showed age, tumor size, tumor invasiveness, and extent of resection were positively related to the post-operative recurrence. In addition, the recurrence rate was also significantly related to the triple invasion (suggesting the tumor invasiveness). For the pathological parameters, E-Ca, Ki67, and β-catenin expressions were also found to be related to the post-operative recurrence. PR expression had no relationship with the post-operative recurrence, which may partially explain female predominance of BM, but not AM. Further multivariate analysis showed only the extent of resection and tumor invasiveness were closely related to the post-operative recurrence of AM. In a more recent study, Ros-Sanjuan et al. showed total resection was the only significant factor associated with recurrence (10). However, even though complete tumor resection is the goal, surgery should be tailored to each patient according to the risks and surgical morbidity (21). In a study involving 76 patients, Kim et al. also found the brain and/or bone involvement predicted an increased risk of treatment failure despite combination therapy (22). Mantle et al. found the cancer cells invaded the brain by advancing along vessels that bridge the gap between the cancer surface and the cortex, and thus they proposed that these cancer cells were the most frequent source of recurrences after “complete” resection (23).

Currently, the use of radiotherapy in meningiomas patients is still controversial. Generally, the decision to use adjuvant radiotherapy is based on the extent of resection and the histologic tumor characteristics, and it is also added in AM and BM patients. Radiotherapy has been used as the primary treatment for non-resectable tumors for decades. In addition, it may serve as a post-operative adjuvant therapy or in case of recurrence for previously resected meningiomas. In a more recent study, results showed radiotherapy was more often applied after incomplete resection, but postoperative radiotherapy did not improve the progression-free survival (PFS) (24). Of note, there are no convincing findings from randomized controlled clinical trials supporting the use of radiotherapy in meningiomas patients. The role for adjuvant radiotherapy in AM is much more controversial compared to that for anaplastic meningiomas. In the present study, the proportion of patients receiving post-operative radiotherapy was comparable between AM patients and BM patients, and radiotherapy was not related to the post-operative recurrence. Our findings were consistent with previous findings although there is benefit from the use of adjuvant radiotherapy, even after complete resection, in AM patients (25). This might be related to the study design of available studies (most retrospective or observational studies). In our study, seven AM patients received post-operative radiotherapy and three developed post-operative recurrence (42.9%), but nine patients had recurrence among 23 patients without post-operative radiotherapy (39.1%). This paradoxical finding might be explained as that patients receiving post-operative radiotherapy usually had incomplete resection. In addition, the small sample size may also be another reason.

There were still limitations in this study. There were only 30 AM patients recruited, and the duration of follow up was relatively short. In addition, only the recurrence was assessed in our study. The retrospective design in this study also limits the expansion of findings in clinical practice. Thus, more randomized, controlled prospective studies are needed to confirm our findings. In addition, in recent years, genetic mutations have been identified as a factor related to the prognosis of meningiomas and they may also be used to guide the treatment of meningiomas (26). It is necessary to investigate the relationship between genetic markers and post-operative recurrence of AM.

Taken together, BM has a higher prevalence in females, and AM patients have a higher post-operative recurrence rate. Among clinicopathological characteristics, the extent of resection and tumor invasiveness are the independent risk factors of post-operative recurrence in AM patients. Thus, to tailor the surgical procedures and maximize the surgical resection will improve the post-operative prognosis of AM patients.

## Data Availability Statement

The datasets generated for this study are available on request to the corresponding author.

## Ethics Statement

The studies involving human participants were reviewed and approved by Institutional Review Board of The First Affiliated Hospital of Medical School of Zhejiang University and Tongde Hospital of Zhejiang Province. Written informed consent for participation was not required for this study in accordance with the national legislation and the institutional requirements.

## Author Contributions

LY and ZR-Y conceived the study design. WY and TD-Z were responsible for patient recruitment. WY, TD-Z, and YX-S organized database and were responsible for statistical calculations. WY, ZR-Y, and LY drafted the article. All authors discussed the results, revised the draft, and approved the final manuscript.

### Conflict of Interest

The authors declare that the research was conducted in the absence of any commercial or financial relationships that could be construed as a potential conflict of interest.
